# The divergent outcome of IL-4Rα signalling on Foxp3 T regulatory cells in listeriosis and tuberculosis

**DOI:** 10.3389/fimmu.2024.1427055

**Published:** 2024-10-17

**Authors:** Julius E. Chia, Robert P. Rousseau, Mumin Ozturk, Sibongiseni K. L. Poswayo, Rodney Lucas, Frank Brombacher, Suraj P. Parihar

**Affiliations:** ^1^ International Centre for Genetic Engineering and Biotechnology (ICGEB), Cape Town Component, Cape Town, South Africa; ^2^ Division of Immunology, Institute of Infectious Diseases and Molecular Medicine (IDM), Department of Pathology, Faculty of Health Sciences, University of Cape Town, Cape Town, South Africa; ^3^ Centre for Infectious Diseases Research in Africa (CIDRI-Africa), Institute of Infectious Diseases and Molecular Medicine (IDM), Faculty of Health Sciences, University of Cape Town, Cape Town, South Africa; ^4^ Research Animal Facility (RAF), Faculty of Health Sciences, University of Cape Town, Cape Town, South Africa; ^5^ Division of Medical Microbiology, Institute of Infectious Diseases and Molecular Medicine (IDM), Department of Pathology, Faculty of Health Sciences, University of Cape Town, Cape Town, South Africa; ^6^ Division of Human Metabolomics, North-West University, Potchefstroom, South Africa

**Keywords:** listeriosis, IL-4Rα, Foxp3 T cells, mice, tuberculosis

## Abstract

**Introduction:**

Forkhead box P3 (Foxp3) T regulatory cells are critical for maintaining self-tolerance, immune homeostasis, and regulating the immune system.

**Methods:**

We investigated interleukin-4 receptor alpha (IL-4Rα) signalling on T regulatory cells (Tregs) during *Listeria monocytogenes* (*L. monocytogenes*) infection using a mouse model on a BALB/c background, specifically with IL-4Rα knockdown in Tregs (Foxp3^cre^IL-4Rα^−/lox^).

**Results:**

We showed an impairment of Treg responses, along with a decreased bacterial burden and diminished tissue pathology in the liver and spleen, which translated into better survival. Mechanistically, we observed an enhancement of the Th1 signature, characterised by increased expression of the T-bet transcription factor and a greater number of effector T cells producing IFN-γ, IL-2 following *ex-vivo* stimulation with heat-killed *L. monocytogenes* in Foxp3^cre^IL-4Rα^-/lox^ mice. Furthermore, CD8 T cells from Foxp3^cre^IL-4Rα^-/lox^ mice displayed increased cytotoxicity (Granzyme-B) with higher proliferation capacity (Ki-67), better survival (Bcl-2) with concomitant reduced apoptosis (activated caspase 3). In contrast to *L. monocytogenes*, Foxp3^cre^IL-4Rα^-/lox^ mice displayed similar bacterial burdens, lung pathology and survival during *Mycobacterium tuberculosis* (*M. tuberculosis*) infection, despite increased T cell numbers and IFN-γ, TNF and IL-17 production.

**Conclusion:**

Our results demonstrated that the diminished IL-4Rα signalling on Foxp3+ T regulatory cells resulted in a loss of their functionality, leading to survival benefits in listeriosis but not in tuberculosis.

## Introduction

T regulatory cells (Tregs) are a subset of CD4 T cells that uniquely express the transcription factor Forkhead box P3 (Foxp3) upon recognition of antigens by T-cell receptors. This expression leads to the suppression of effector T-cell responses, making Tregs pivotal in the maintenance of immune homeostasis (1). Tregs with a reduction and/or mutation in Foxp3 lead to various autoimmune diseases and immune dysregulation in both mice and humans (2). The breadth of their receptors, costimulatory molecules, and cytokine milieu affects their functions at large. For instance, Tregs expressing CTLA-4 cause trans-endocytosis of CD80/CD86, resulting in impaired CD28 costimulation, thus impeding T-cell responses (3). Tregs delay T-cell priming by hindering antigen presentation through direct interaction with peptide-MHC complex (pMHC) and by removing this complex from the surface of the antigen-presenting cells (4). Tregs also modulate anti-inflammatory responses through the secretion of interleukin (IL)-10 and tumor growth factor beta (TGF-β) (5, 6).

The cytokine milieu of Th1 (interferon (IFN)-γ and IL-12) or Th2 (IL-4) responses modulates Treg cell development and function (7–9). IL-4 signals through the IL-4 receptor alpha (IL-4Rα), which is associated with archetypal type 2 immunity. IL-4Rα is expressed on both innate and adaptive immune cells, including Tregs, where it controls Foxp3 expression (10, 11) and promotes reprogramming toward the Th2 phenotype via the IL-4Rα-STAT6 axis (12), suggesting that IL-4Rα plays a restrictive role in Treg functions.

*Listeria monocytogenes* (*L. monocytogenes*), a gram-positive bacteria, is the causative agent of listeriosis. It is a foodborne intracellular pathogen that infects a wide range of cells, including neutrophils and macrophages, which are required for host protection (13, 14). In the 1980s, a series of outbreaks occurred in Europe and the USA (15–17), with more recent cases reported in South Africa (2017–2018) (18). Despite the availability of effective antibiotics, the mortality rate remains at 30% (19). During pregnancy, Tregs expand to maintain maternal tolerance to the foetus; however, this expansion is associated with greater susceptibility to *L. monocytogenes* (20). In *Mycobacterium tuberculosis* (*M. tuberculosis*) infection, Tregs expand, which delays host protective effector T-cell responses and facilitates the establishment of early infection in the lungs (21, 22).

Here, we demonstrated that the deletion of IL-4Rα signalling on Tregs resulted in decreased Foxp3 Tregs and increased survival rates of mice in listeriosis. These observations were accompanied by decreased tissue bacterial loads and liver pathology, attributable to enhanced CD8 T-cell immune responses and cytotoxic functions. In contrast, IL-4Rα-deficient Tregs did not affect the outcome of tuberculosis, despite increased CD4 T-cell responses, reflecting the differential roles of IL-4Rα signalling in Tregs during bacterial infections.

## Methods

### Mice

Deletion of the *Il4rα* gene in Foxp3-expressing cells (Foxp3^cre^IL-4Rα^−/lox^) was generated and characterized in BALB/c background mice, as previously described (23). Mice were housed in the specific pathogen-free (SPF) animal facility of the Faculty of Health Sciences, University of Cape Town. All mice used in the experiments were aged 8–12 weeks and sex-matched.

### *Listeria monocytogenes* and *Mycobacterium tuberculosis* infections

*L. monocytogenes* (virulent EGD-e strain) was cultured and maintained as previously described (24). Foxp3^cre^IL-4Rα^−/lox^ and littermate controls were infected via intraperitoneal injection with 200 µl of phosphate buffered saline (PBS) using an insulin syringe containing 2 × 10^5^ colony forming unit (CFU) of *L. monocytogenes* for survival studies, and with 2 × 10^4^ for time course experiments.

*M. tuberculosis* H37Rv was grown in Middlebrook 7H9 broth and passaged in mice to maintain virulence. The mice were infected via aerosol inhalation, mimicking the natural route of *M. tuberculosis* infection, as previously described (25). A total of 2 × 10^6^/ml of live bacteria was suspended in 6 ml of PBS solution to obtain a low dose of 100 CFU/lung using a glass-col nebulising aerosol inhalation system. The infection dose was confirmed by plating lung homogenates from three to five mice on 7H10 Middlebrook plates 24 h after infection.

### Determination of bacterial burdens

For *L. monocytogenes* and *M. tuberculosis* infections, bacterial loads were determined at various time points. Organs were collected aseptically and homogenised in PBS containing 0.05% Tween 80. Homogenates were serially diluted 10-fold in PBS, and 100 µl was plated on tryptic soy agar plates for *L. monocytogenes* and on 7H10 agar plates for *M. tuberculosis.* Plates were incubated at 37°C for 24 h for *L. monocytogenes*, and for 21 days for *M. tuberculosis* for colony counting.

### Tissue histopathology

Consistent lobes of the spleen or liver were collected from *L. monocytogenes*-infected animals, while lungs were collected from *M. tuberculosis-*infected animals. The sections were fixed with 4% formalin and rehydrated with xylol and alcohol during preparation of slides. Three different cuts (2–3 µm) were obtained from each mouse for hemotoxylin and eosin (H&E) staining to observe pathological changes, immune cell infiltration, and lesion sizes. Images were acquired using a Nikon Eclipse 90i Microscope. Analysis and visualization of alveolar air spaces were determined using the Nikon NIS element software.

### Immune response in tissue homogenates

Concentrations of the various cytokine and chemokines (IL-1α, IL-1β, IL-4, IL-6, IL-10, IL-12p40, IL-12p70, IL-17, IL-23, IFN-γ, IFN-β, TGF-β, tumor necrosis factor (TNF), granulocyte-macrophage colony-stimulating factor (GM-CSF), macrophage colony-stimulating factor (M-CSF), C-C motif ligand 2 (CCL2), C-C motif ligand 3 (CCL3), C-X-C motif ligand 1 (CXCL1), C-X-C motif ligand 2 (CXCL2), C-X-C motif ligand 5 (CXCL5), and C-X-C motif ligand 10 (CXCL10)) were determined in tissue homogenates or culture supernatants using ELISA along with nitrite concentrations by Griess assay, as determined previously (25).

### Isolation and stimulation of mediastinal lymph node cells

The mediastinal lymph node was harvested from mice, mechanically digested using syringe plungers, and passed through 70 µm and then 40 µm strainers to prepare single-cell suspensions. Cells were centrifuged at 1,200 rpm for 10 min at 4°C. Single cells were resuspended in 2–5 ml of complete Dulbecco’s modified Eagle’s medium (DMEM, Gibco, NY, USA) supplemented with 10% FCS, and viable cells were counted with Trypan Blue (0.4%). A total of 2 × 10^6^ cells were seeded in 100 µl of media and left unstimulated or stimulated with H37Rv lysate (10 µg/ml), or phorbol-12-myristate-13-acetate (PMA) (50ng/ml)/ionomycin (250 ng/ml) with monensin (200 μM) for 8 h.

### Flow cytometry

Single-cell suspensions were prepared as previously described (25). Briefly, 1 × 10^6^ cells from liver and spleen tissues for *L. monocytogenes*, as well as lung and lymph node tissues for *M. tuberculosis*, were stained with rat anti-mouse IL-4Rα (BD Biosciences, NJ, USA) along with other markers to determine the populations of the following cell types: dendritic cells (CD11b^+^CD11c^+^MHCII^+^), spleen macrophages (CD11b^+^CD11c^-^Ly6C^−^), liver macrophages (CD11b^low^F4/80^+^), lung alveaolar macrophages (CD64^+^MerTK^+^SiglecF^+^CD11c^+^), neutrophils (Ly6G^+^CD11b^+^), eosinophils (SiglecF^+^CD11b^+^CD64^−^), CD4 T cells (CD19^−^CD3^+^CD4^+^CD8^−^), CD8 T cells (CD19^−^CD3^+^CD4^−^CD8^+^), CD4/CD8 Naïve (CD62L^+^CD44^−^), memory (CD62L^+^CD44^+^), and effector (CD62L^+^CD44^+^). All antibodies were purchased from BioLegend, San Diego, USA unless otherwise stated. The staining was performed using an antibody mix (50 μl) containing rat serum (2%) and FcγR blocking antibody (10 µg/ml) in PBS supplemented with 1% BSA and 0.1% NaN_3_.

Cytokines were assessed by intracellular cytokine staining (ICS) as previously described (23), using antibodies including anti-IL-2-FITC, IFN-γ-A700, IL-4-PE, IL-17-PerCP Cy5.5, and IL-10-FITC. Intranuclear staining was performed similarly to ICS with eBioscience, San Diego, USA Foxp3/transcription factor staining buffer, utilizing antibodies such as anti-Foxp3-APC, Ki-67-PE, Bcl-2-FITC, Caspase3-PE, GranzymeB-BV421, and GATA3-PerCP Cy5.5. The acquisition was achieved using the LSRFortessa™. The gating strategy is presented in **Supplementary Figures S1**–**S4**. All generated data from the Fortessa were analysed using Flowjo software (FlowJo v10.0.7).

### Real-time PCR

Total RNA was extracted from splenocytes and hepatocytes following *L. monocytogenes* infection using the Qiagen RNeasy Mini Kit, in accordance with the manufacturer’s protocol. RNA quality was determined using NanoDrop 2000. cDNA was synthesized through reverse transcription using first-stranded cDNA Synthesis Kit (Roche, Basel, Switzerland), employing random hexamer primer and anchored oligo dT primers. Quantitative real-time PCR was performed using LightCycler^®^ 480 SYBR Green I Master Mix in LightCycler^®^ 480 II (Roche). *Hprt* served as the housekeeping gene for absolute quantifications.

### Publicly available human TB transcriptomics datasets

Transcriptional signatures of *il4ra and foxp3* were analyzed from prospective cohorts of active TB cases and Quantiferon-positive latent TB (LTBI) cases in the publicly available whole blood gene expression dataset (GSE19442) from South Africa (26). Transcriptional profiles in the whole blood of participants with tuberculosis undergoing treatment were plotted and analyzed from another publicly available South African cohort dataset (GSE40553) (27).

### Statistics

Data were analysed using GraphPad Prism software (v6.0 GraphPad Software, La Jolla, CA, USA). The statistical tests employed were either Student’s *t*-test (two-tailed with unequal variance) or one-way ANOVA with Dunnett’s *post-hoc* test when comparing more than two groups. A *p value of less than 0.05 was considered significant, depicting **p < 0.01, ***p < 0.001 and ****p < 0.0001.

## Results

### IL-4Rα and Foxp3 mRNA expression changes differentially in early and late *L. monocytogenes* infection in mice

**Figure 1:**
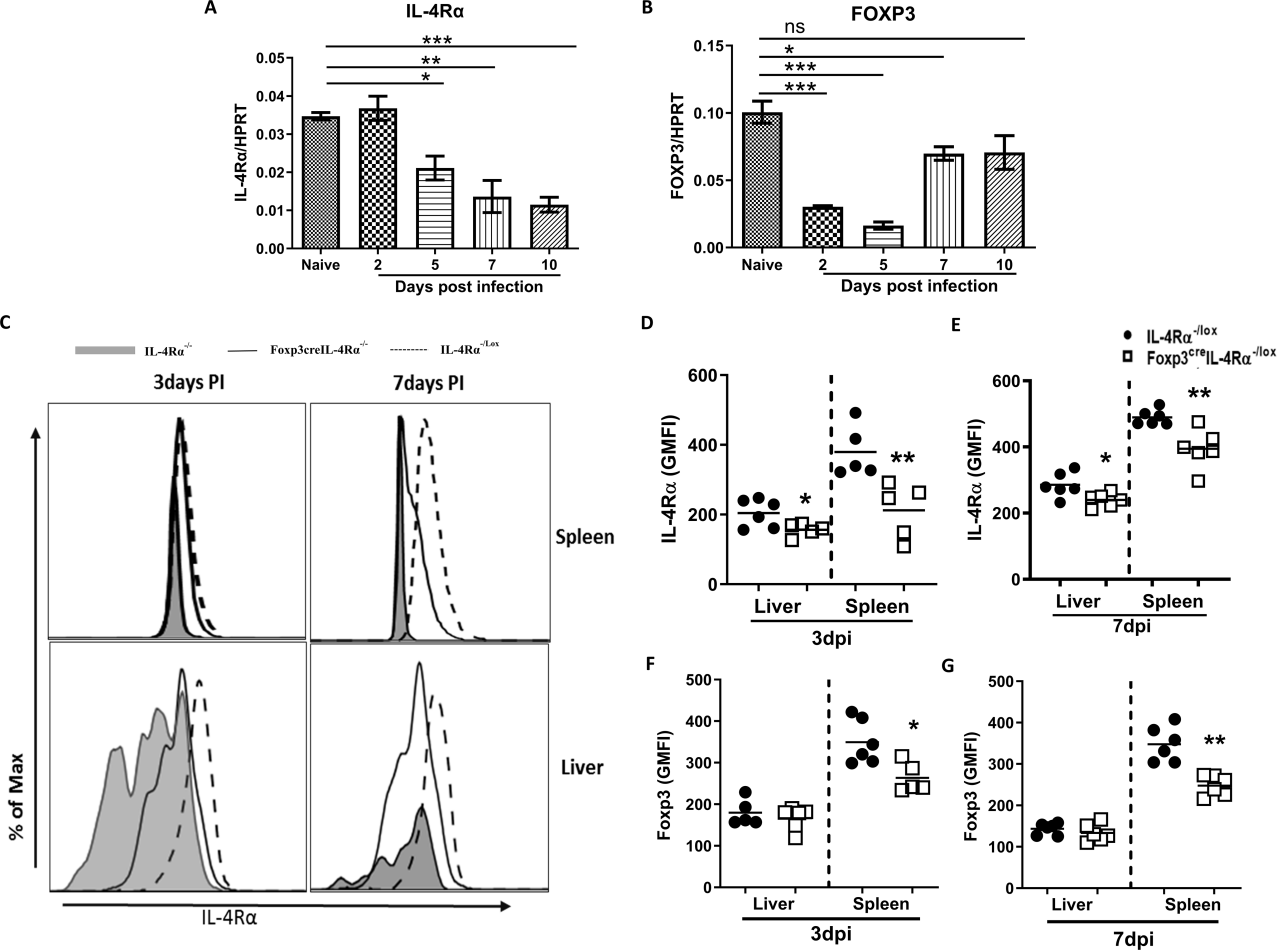
Listeria infection decreases mRNA expression of *IL4rα* and *Foxp3* in the spleen. Mice were infected via an intraperitoneal route with 2 × 10^4^ CFU of *L. monocytogenes*. At the indicated days, mice were euthanized to measure **(A)** *Il4rα* **(B)** *Foxp3* mRNA levels, normalized to the *Hprt* housekeeping gene in the spleen. Single-cell suspension from the liver and spleen was analysed by flow cytometry to determine IL-4Rα expression on Foxp3 Tregs. **(C)** Histogram representation of IL-4Rα^−/−^ (grey shaded), Foxp3^cre^IL-4Rα^−/lox^ (solid line), and IL-4Rα^−/lox^ (dashed line) tissues, along with IL-4Rα geometric mean fluorescence intensity (GMFI) quantification in the spleen and liver at **(D)** 3 and **(E)** 7 dpi. Foxp3 expression on CD4^+^ T cells is represented as GMFI values at **(F)** 3 and **(G)** 7 dpi. Data are represented as mean ± SEM, representative of two independent experiments with **(A, B)** *n* = 3 mice/time point and **(C–G)** *n* = 5–7 animals, analysed using a two-tailed unpaired Student’s *t*-test (^*^ *p* < 0.05; ^**^ *p* < 0.01; ^***^ *p* < 0.001).

To determine whether *L. monocytogenes* infection regulates the expression of IL-4Rα and Foxp3, we assessed mRNA expression in the spleen over the course of 10 days in BALB/c mice infected with a sublethal dose 2 × 10^4^ CFU of *L. monocytogenes* intraperitoneally. At 5, 7, and 10 days after infection, *Il4rα* mRNA transcript levels were gradually decreased in the spleen (**Figure 1A**), suggesting that *L. monocytogenes* infection downregulates *Il4rα* expression. Foxp3 transcript levels also decreased significantly on days 2 and 5 (**Figure 1B**). Foxp3 expression was restored on day 10, although the levels were similar to those observed on day 7; greater variation in the data resulted in no significant difference on day 10 (**Figure 1B**). The increase in Foxp3 expression at this later stage is expected, as mice typically recover from *L. monocytogenes* infection by day 10 (28). Given the differences in IL-4Rα transcript levels, we investigated the biological role of the receptor *in vivo* using mice that lack IL-4Rα specifically on Foxp3 Tregs (Foxp3^cre^IL-4Rα^−/lox^). These mice were intraperitoneally infected with 2 × 10^4^ *L. monocytogenes* and euthanized 3 and 7 days postinfection. We found a significant reduction in IL-4Rα surface protein levels in both the spleen and liver of Foxp3^cre^IL-4Rα^−/lox^ mice compared to littermate controls at 3 and 7 days postinfection (dpi) (**Figures 1C-E**), with IL-4Rα^−/−^ mice used as additional controls. Moreover, we confirmed cell-specific deletion of the receptor on Foxp3 Tregs in both organs during the course of infection, as expected, though levels did not reach those found in IL-4Rα null mice. This is due to the limitations of the Cre-lox system, where the specificity of Cre expression depends on the fidelity of promoters, which may result in partial gene deletion (see gating strategy **Supplementary Figure S1**). The characterisation of Foxp3^cre^IL-4Rα^−/lox^ mice had been previously published by our laboratory (23). During *L. monocytogenes* infection, the expression of Foxp3 is significantly decreased in CD4 T cells in the spleen, but not the liver, which could be due to the fewer Foxp3 cells in the liver at 3 and 7 dpi (**Figures 1F, G**). These results suggest that Foxp3 expression in CD4 T cells is decreased in the absence of IL-4Rα signalling during *L. monocytogenes* infection.

### Foxp3^cre^IL-4Rα^−/lox^ mice showed enhanced survival during *L. monocytogenes* infection

**Figure 2:**
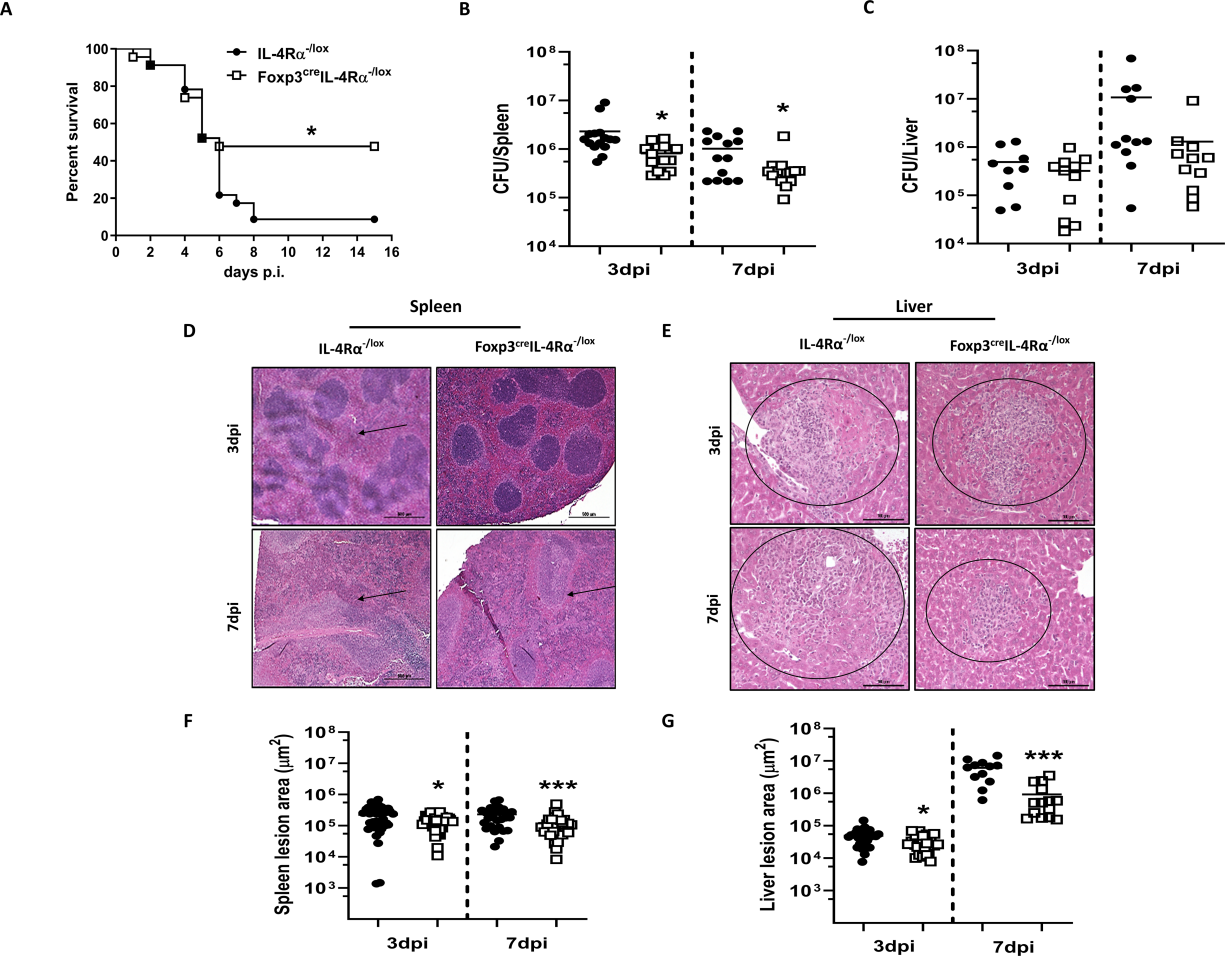
Foxp3^cre^IL-4Rα^−/lox^ mice showed increased survival and decreased tissue burdens and pathology. **(A)** Survival of Foxp3^cre^IL-4Rα^−/lox^ and littermate control animals over 15 days following infection with 2 × 10^5^ CFU of *L. monocytogenes*. **(B)** Spleen and **(C)** liver bacterial burden was determined at 3 and 7 days postinfection. At 3 and 7dpi, spleen and liver tissues were formalin-fixed and stained with H&E for histopathological analysis. Representative sections of the **(D)** spleen and **(E)** liver with arrows showing white pulp atrophy and circles indicating cellular infiltration. Three 30-µm apart cuts per tissue were analysed (scalebar = 100 µm; magnification, × 200). **(F, G)** Quantification of splenic atrophic areas and liver lesion size. Data are represented as mean ± SEM of **(A)** 8–10 mice/group, analysed using Mantel–Cox test (*p* = 0.0123), and **(B–G)** 10–15 mice/group from pooled data across three independent experiments, analysed using a two-tailed unpaired Student’s *t*-test (^*^ *p* < 0.05; ^***^ *p* < 0.0001).

Given the reduced expression of Foxp3 in Tregs during *L. monocytogenes* infection (**Figure 1**), we investigated the effect of IL-4Rα deletion on Foxp3 Treg cells in relation to host survival and bacterial burden. Mice were infected intraperitoneally with a lethal dose (LD_50_) of *L. monocytogenes* (2 × 10^5^ CFU/mouse). Foxp3^cre^IL-4Rα^−/lox^ mice showed enhanced survival in comparison to control littermates during *L. monocytogenes* infection (**Figure 2A**). To better understand this survival benefit, we infected Foxp3^cre^IL-4Rα^−/lox^ mice with a sublethal dose of *L. monocytogenes* (2 × 10^4^ CFU/mouse) for time-kinetic experiments. At 3 and 7 dpi time points, mice displayed a significant reduction in the listerial burden in the spleen (**Figure 2B**), while liver burdens remained unaffected (**Figure 2C**). We performed hematoxylin and eosin staining (H&E) to evaluate the histopathology of the spleen and liver. At 3 and 7 dpi, Foxp3^cre^IL-4Rα^−/lox^ mice displayed smaller lesion size, as indicated by the atrophic white splenic pulp (**Figure 2D**), and decreased lesion size in the liver (**Figure 2E**) compared to littermate control IL-4Rα^−/lox^ animals. This was further confirmed following the quantification of the lesion sizes in both the spleen and liver, although the liver had exhibited similar bacterial burdens (**Figures 2F, G**). This suggests that the deletion of IL-4Rα on T regulatory cells resulted in decreased splenic burdens, reduced cellular infiltration into the tissues, and consequently smaller lesion sizes during *L. monocytogenes* infection.

### Increased proinflammatory cytokines in Foxp3^cre^IL-4Rα^−/lox^ mice during *L. monocytogenes* infection

**Figure 3:**
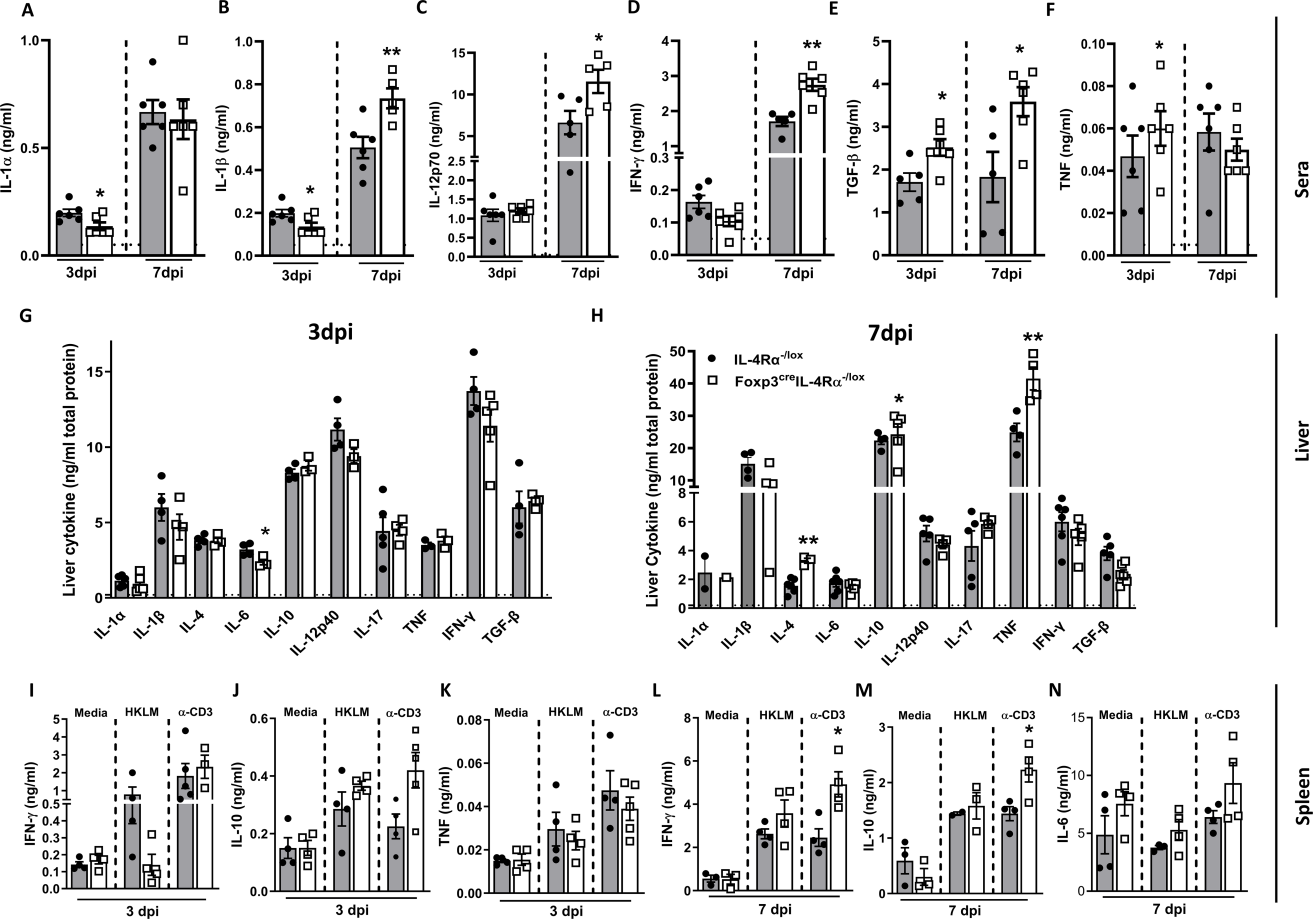
Foxp3^cre^IL-4Rα^−/lox^ mice showed increased proinflammatory cytokines in sera and spleen. Cytokine concentrations were measured in the serum, liver, and supernatants of restimulated splenocytes by ELISA. **(A)** IL-1α, **(B)** IL-1β, **(C)** IL-12p70, **(D)** IFN-γ, **(E)** TGF-β, and **(F)** TNF in serum; **(G, H)** cytokines in liver homogenates at 3 and 7 dpi. Spleen cells were restimulated with α-CD3 and heat-killed *L. monocytogenes* (HKLM) or left unstimulated for 72 h to determine **(I)** IFN-γ, **(J)** IL-10, and **(K)** TNF in the supernatants by ELISA at 3 dpi, and **(L)** IFN-γ, **(M)** IL-10, and **(N)** IL-6 at 7 dpi. Data are representative of mean ± SEM from two individual experiments and analysed using a two-tailed unpaired Student’s *t*-test (^*^ *p <* 0.05; ^**^ *p* < 0.01).

We next assessed whether the humoral immune response was differentially affected in Foxp3^cre^IL-4Rα^−/lox^ mice during *L. monocytogenes* infection. At 3 dpi, serum analysis revealed a decrease in IL-1α (**Figure 3A**) and IL-1β (**Figure 3B**), along with an increase in the regulatory cytokine TGF-β (**Figure 3E**). At 7 dpi, we found a significant increase in IL-1β (**Figure 3B**), IL-12p70 (**Figure 3C**), IFN-γ (**Figure 3D**), and TGF-β (**Figure 3E**) in Foxp3^cre^IL-4Rα^-/lox^ mice, while TNF levels remained unchanged (**Figure 3F**). We then analysed the cytokine response in liver homogenates. At 3 dpi, there was a significant decrease in IL-6 (**Figure 3G**). In contrast, at 7 dpi, there was a significant increase in TNF, IL-10, and IL-4, suggesting a dampening effect in the absence of IL-4Rα on Foxp3 Tregs; however, there were no differences in IL-17, IFN-γ, TGF-β, IL-12p40, and IL-6 (**Figure 3H**). The similar listerial burden in the liver may account for no major changes or the shift in inflammatory–regulatory cytokine landscape balance in the immune response. We next examined the spleen cytokine profile following restimulation with either heat-killed *L. monocytogenes* (HKLM) or anti-CD3 or left unstimulated. At 3 dpi, there were no major differences in the production of IFN-γ, IL-10, and TNF (**Figures 3I-K**). However, at 7 dpi, there was a significant increase in IFN-γ and IL-10 following anti-CD3 stimulation (**Figures 3L-N**). The modest increase in IFN-γ may contribute to the decreased bacterial burdens, as IFN-γ plays a crucial role in the clearance of *L. monocytogenes* (29). Altogether, IL-4Rα signalling on Foxp3 Tregs modulates the immune response in mice during *L. monocytogenes* infection.

### Absence of IL-4Rα signalling on Foxp3 alters immune cell populations early after *L. monocytogenes* infection

**Figure 4:**
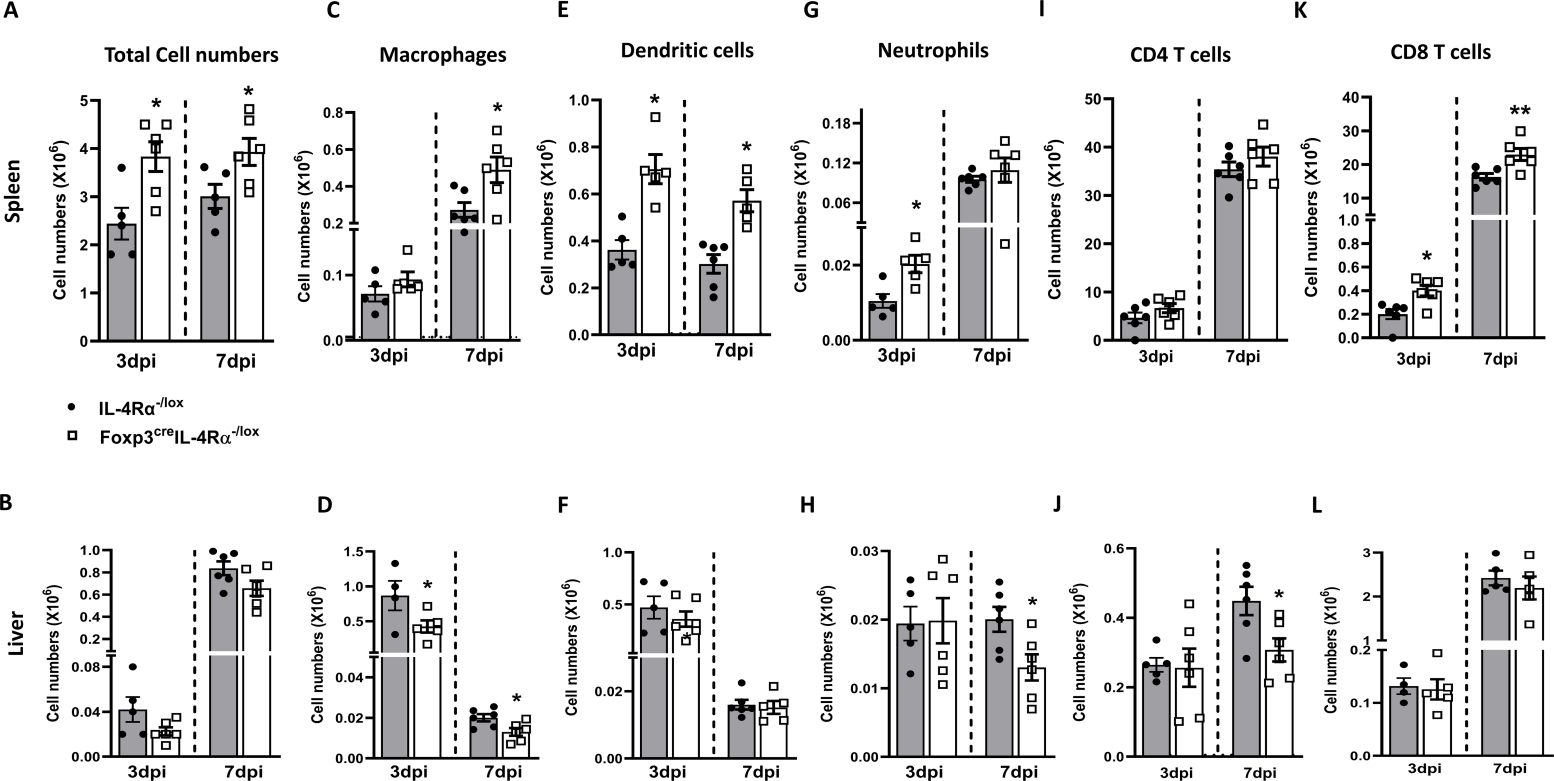
Foxp3^cre^IL-4Rα^−/lox^ mice showed increased myeloid cell populations and CD8 T cells in the spleen during *L. monocytogenes* infection. Single-cell suspension of the spleen and liver was subjected to surface staining to determine myeloid and lymphoid populations using BD Fortessa at 3 and 7 dpi. Total cell numbers in the **(A)** spleen and **(B)** liver. **(C, D)** Macrophage, **(E, F)** Dendritic cell, **(G, H)** Neutrophil, **(I, J)** CD4 T cells, and **(K**, **L)** CD8 T-cell numbers are depicted. Data are represented as mean ± SEM of *n* = 6–8 mice from two independent experiments and analysed using two-tailed unpaired Student’s *t*-test (^*^ *p <* 0.05; ^**^ *p* < 0.01).

To understand which immune cell subsets were involved in the augmented infiltration, cell populations were analysed in the liver and spleen. There was a significant increase in the total cell number harvested from the Foxp3^cre^IL-4Rα^−/lox^ mice compared to littermate controls in the spleen at 3 and 7 dpi (**Figure 4A**); however, cell numbers were unaffected in the liver (**Figure 4B**). Additionally, there was a significant increase in macrophages in the spleen of Foxp3^cre^IL-4Rα^−/lox^ mice at 7 dpi (**Figure 4C**), while macrophages were decreased in the liver at both time points (**Figure 4D**). The number of dendritic cells (DCs) in the spleen also increased at both 3 and 7 dpi in the Foxp3^cre^IL-4Rα^−/lox^ mice (**Figure 4E**), but no differences were observed in the liver (**Figure 4F**). There was an increase in neutrophils early at 3 dpi in the spleen (**Figure 4G**) and a decrease in the liver at 7 dpi in the Foxp3^cre^IL-4Rα^−/lox^ mice (**Figure 4H**). Neutrophils are known to control *L. monocytogenes* during the early stages of infection, which could explain the decreased splenic burden in the Foxp3^cre^IL-4Rα^−/lox^ mice (30). We also evaluated T cells, given their important role in *L. monocytogenes* infection (31–33). T-cell populations in the liver and spleen were investigated at both 3 and 7 dpi. CD4 T-cell numbers in the spleen and liver were similar for both organs (**Figures 4I, J**). However, a significant increase in the CD8 T-cell population was observed in the spleen (**Figure 4K**), but not in the liver (**Figure 4L**). Thus, IL-4Rα signalling on Foxp3 influences myeloid populations and CD8 T cells to a greater extent in the spleen during *L. monocytogenes* infection in mice.

### Foxp3^cre^IL-4Rα^−/lox^ mice showed enhanced T-cell-mediated effector phenotype during *L. monocytogenes* infection

**Figure 5:**
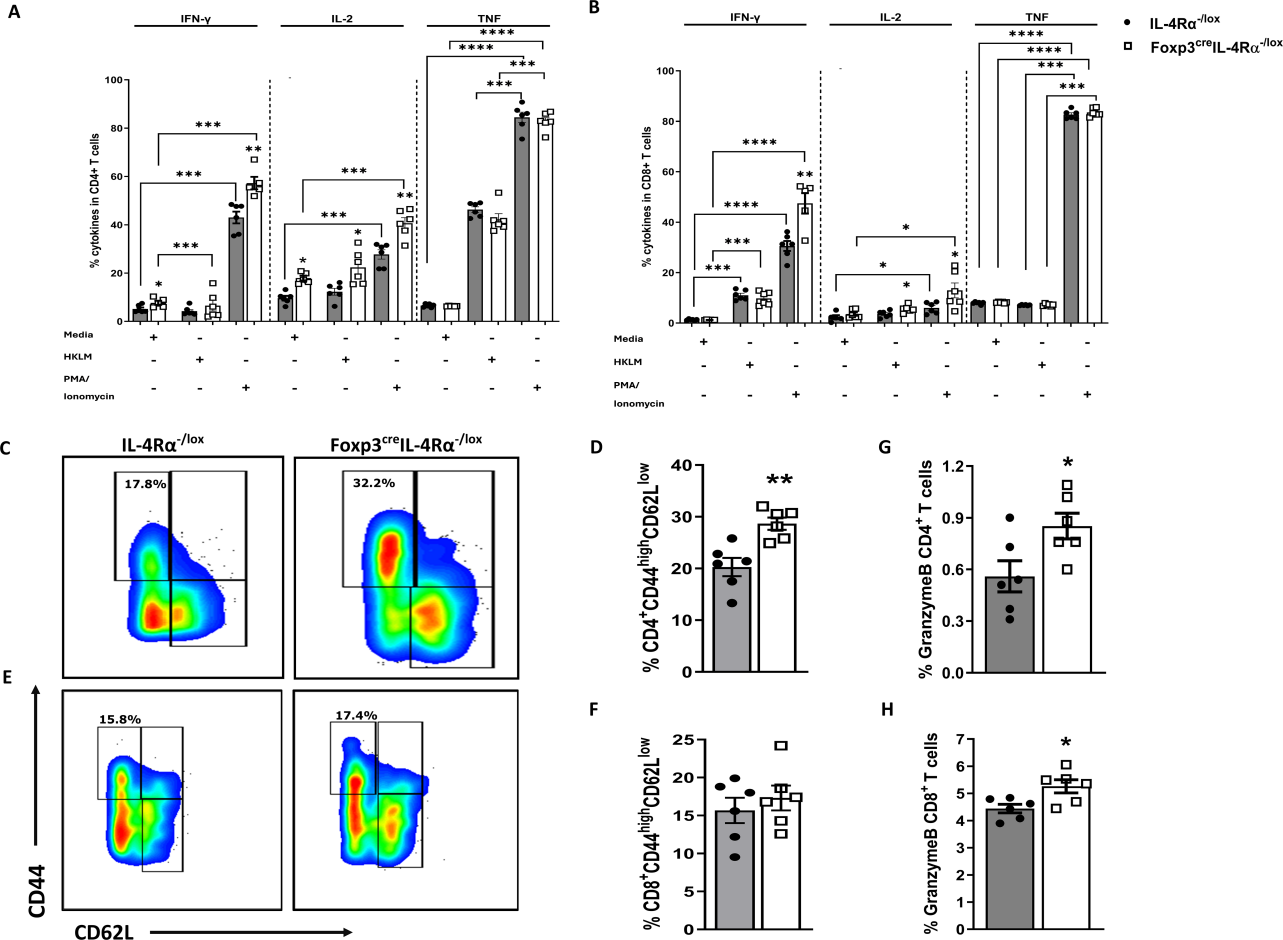
Enhanced splenic CD4 T-cell effector function in Foxp3^cre^IL-4Rα^−/lox^ mice during *L. monocytogenes* infection. Single-cell suspensions were prepared from spleens of 7-day infected mice, and 2 million cells were seeded in media, PMA/ionomycin, and heat-killed *L. monocytogenes* stimulation for 6 h, followed by monensin blockage to perform intracellular cytokine staining in **(A)** CD4 and **(B)** CD8 T cells. **(C, D)** Representative flow plots and percentages of effector T-cell population pregated on CD4 T cells. **(E, F)** Representative flow plots and percentages of effector T-cell population pregated on CD8 T cells. Granzyme B expression in **(G)** CD4 and **(H)** CD8 T cells are shown. Data are represented as mean ± SEM of *n* = 6–8 mice/time point across two independent experiments, analysed using a two-tailed unpaired Student’s *t*-test (^*^ *p <*0.05; ^**^ *p <*0.01; ***p < 0.001; ****p < 0.0001).

CD8 T cells are crucial for the clearance of *L. monocytogenes* (34, 35). Their increased numbers in Foxp3^cre^IL-4Rα^−/lox^ mice are quite notable, prompting further analysis of these cells to understand the secretion of other intracellular cytokines and granzyme B, which are produced mainly by CD8 cytotoxic T cells. We sought to elucidate the underlying mechanisms behind the enhanced phenotype in the spleen at 7 dpi. To this end, we infected mice with 2 × 10^4^ low-dose CFUs of *L. monocytogenes* and euthanized at 7 dpi. Considering that CD8 cytotoxic T cell is one of the predominant cellular populations responsible for killing *L. monocytogenes* (36), we restimulated spleen cells with HKLM, PMA/ionomycin, or left unstimulated to perform intracellular cytokine staining for IFN-γ, IL-2, TNF, and granzyme B in both CD4 and CD8 T cells. In unstimulated cells, there was a significant increase in the production of IFN-γ and IL-2 by CD4 T cells in Foxp3^cre^IL-4Rα^−/lox^ mice (**Figure 5A**), but not in CD8 T cells (**Figure 5B**). Stimulation with HKLM also led to increase in IL-2 but not in IFN-γ and TNF in both the CD4 (**Figure 5A**) and CD8 T cells (**Figure 5B**). As expected, stimulation with PMA/ionomycin led to a significant increase in the production of IFN-γ, IL-2, and TNF levels when compared to unstimulated cells in both CD4 (**Figure 5A**) and CD8 (**Figure 5B**) T cells of Foxp3^cre^IL-4Rα^−/lox^ mice.

We next assessed whether CD4 and CD8 T cells had an augmented effector phenotype. Foxp3^cre^IL-4Rα^−/lox^ mice showed significantly higher CD4 T-cell (**Figures 5C, D**) but not CD8 T-cell (**Figures 5E, F**) effector/effector memory phenotypes (CD44^high^CD62L^low^) compared to littermate controls at 7 dpi, which may induce IFN-γ production, a key Th1 response cytokine. Granzyme B is an important *L. monocytogenes* host-killing protease produced by cytotoxic cells, thus we assessed whether the deletion of IL-4Rα signalling in Tregs affected the ability of T cells to produce granzyme B. Granzyme B is essential for killing of *L. monocytogenes* (37) and is produced largely by CD8 T cells, although activated CD4 T cells can also produce it (38). We observed a significant increase in the production of granzyme B in both CD4 (**Figure 5G**) and CD8 (**Figure 5H**) T cells of Foxp3^cre^IL-4Rα^−/lox^ mice. Collectively, these data suggest that the deletion of IL-4Rα signalling on Foxp3 cells leads to augmented cytotoxic granzyme B production and an effector T-cell phenotype that promotes the production of IFN-γ and IL-2 cytokines.

### The deletion of IL-4Rα on Foxp3 Tregs enhanced T-bet expression in T cells

**Figure 6:**
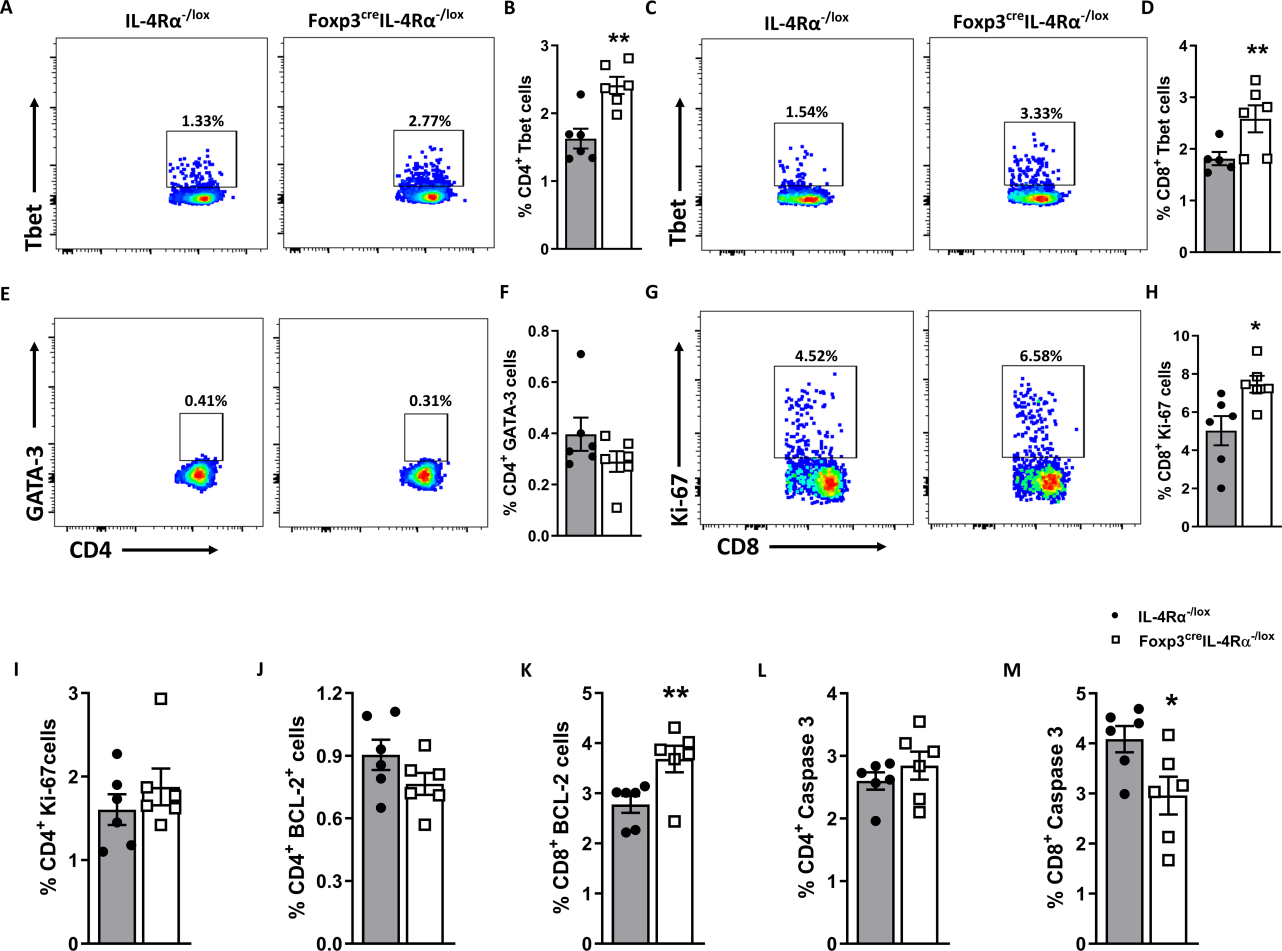
Splenic CD8 T cells expand and express higher T_bet_ in Foxp3^cre^IL-4Rα^−/lox^ mice duirng *L. monocytogenes* infection. A single-cell suspension of splenocytes was stained for intranuclear transcription factors T-bet and GATA-3 from mice euthanized at 7 dpi. Representative flow plots and frequencies of T-bet^+^ in **(A, B)** CD4 and **(C, D)** CD8 T-cell populations. **(E, F)** Representative flow plots and frequencies of GATA-3^+^ CD4 T cells. **(G, H)** Representative flow plots and frequencies of Ki-67^+^ CD8 and **(I)** Ki-67^+^CD4 T cells. **(J, K)** BCL-2^+^ CD4 and CD8 T cells and **(L**, **M)** Caspase 3^+^ CD4 and CD8 T cells are shown. Grey bars represent IL-4Rα^−/lox^ mice, and white bars represent Foxp3^cre^IL-4Rα^−/lox^ mice. Data are represented as mean ± SEM of *n* = 6–8 mice/time point from two independent experiments, analysed using a two-tailed unpaired Student’s *t*-test (^*^ *p <*0.05; ^**^ *p <*0.01).

To delineate the effector phenotype function, we assessed the expression of transcription factor T-bet in T helper and cytotoxic T cells. The T-bet expression is known to regulate the Th1 phenotype and the production of cytokines such as IFN-γ, IL-2, and TNF (39). Remarkably, the absence of IL-4Rα signalling on Foxp3 T regulatory cells resulted in a significant increase in the percentage of T-bet expression in both CD4 (**Figures 6A, B**) and CD8 (**Figures 6C, D**) T cells following *L. monocytogenes* infection at 7 dpi. The T-bet transcription factor is known to promote the growth and differentiation of the Th1 subset while concomitantly blocking the other subsets (40, 41). However, the increase in T-bet expression did not come at the expense of GATA3 expression (**Figures 6E, F**), which remained unaffected in Foxp3^cre^IL-4Rα^−/lox^ mice. These results suggest a shift toward a Th1 phenotype in the absence of IL-4Rα expression on Foxp3 T regulatory cells during *L. monocytogenes* infection.

### The absence of IL-4Rα on Foxp3 Tregs enhanced CD8 T-cell proliferation and survival

It has been reported that infection with *L. monocytogenes* causes the destruction of the white pulp by apoptosis *in vivo* (42). To understand the augmented histopathological destruction of white splenic pulp in the Foxp3^cre^IL-4Rα^−/lox^ at 7 dpi, we investigated activated caspase 3 expression, a marker of apoptosis, along with anti-apoptotic B-cell lymphoma-2 (Bcl-2) expression, which inhibits apoptosis (43). Therefore, we evaluated the deletion of IL-4Rα deletion on Tregs and its effect on the apoptosis and survival of CD8 T cells. The presence of Bcl-2 and Ki-67 markers indicates cell proliferation and nuclear protein activity during cell division. We investigated CD8 T-cell proliferation and observed a significant increase in Ki-67 (**Figures 6G, H**) and BCL-2 (**Figure 6K**), along with a corresponding decrease in caspase 3 (**Figure 6M**) in Foxp3^cre^IL-4Rα^−/lox^ mice. However, no differences in CD4 T cells expressing Ki-67 (**Figure 6I**), BCL-2 (**Figure 6J**), and caspase 3 (**Figure 6L**) were observed. A smaller population of CD8 memory T cells implies that fewer T cells are long-lived and capable of providing protection during subsequent infections (44). These results suggest that in the absence of IL-4Rα on Foxp3 Tregs, CD8 T cells expand, experience decreased apoptosis, and exhibit better survival.

### IL-4Rα transcript levels increased in active TB and reverted back after the completion of treatment

**Figure 7:**
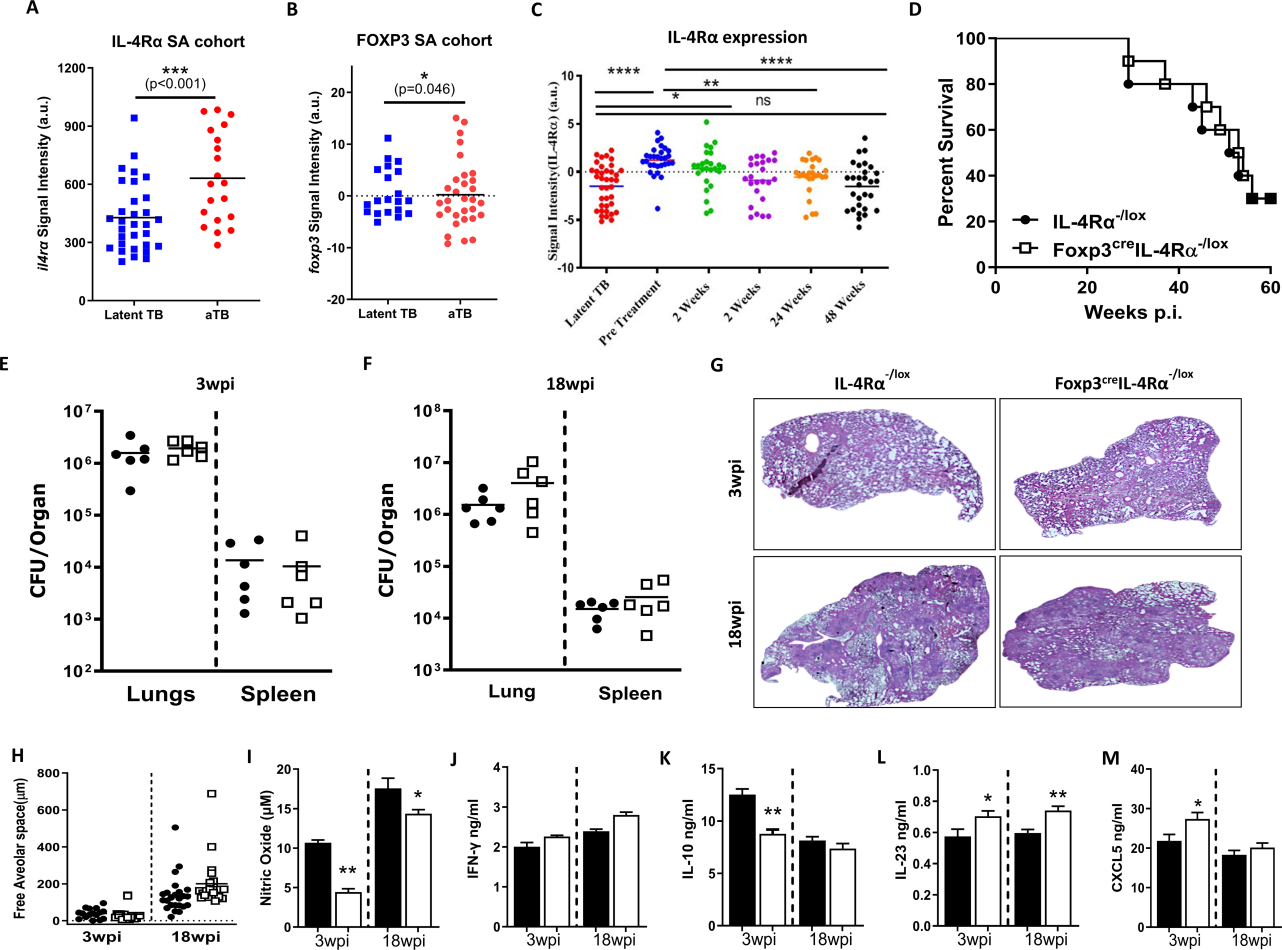
Foxp3^cre^IL-4Rα^−/lox^ mice exhibited comparable TB disease outcomes to littermate controls. **(A)** *Il4rα* expression and **(B)** *Foxp3* expression in active TB and latent TB individuals. **(C)** *Il4rα* expression in a longitudinal cohort until the completion of anti-TB treatment, using whole blood data from a publicly available dataset. Control littermates (IL-4Rα^−/lox^) and Foxp3^cre^ IL-4Rα^−/lox^ mice were infected by aerosol with 100 CFU/mouse of *M. tuberculosis* H37Rv. **(D)** Mortality after 65 weeks postinfection (*n* = 9–10 mice/group). **(E, F)** The bacterial burden in the lungs and spleen at 3 and 18 weeks postinfection (*n* = 5–6 mice/group) and **(G, H)** Representative image of H&E histopathology staining and quantification of alveolar spaces at the indicated time points. Lung homogenates were analysed for the indicated cytokines by ELISA: **(I)** Nitrite oxide (NO), **(J)** IFN-γ, **(K)** IL-10, **(L)** IL-23, and **(M)** CXCL5. Data represented as mean ± SEM of signal intensity (a.u.) were plotted and analysed by **(A, B)** unpaired Student’s *t*-test, **(C)** one-way ANOVA with Turkey’s multiple comparison test, **(D)** Mantel–Cox test, and **(E**–**M)** unpaired Student’s *t*-test. The data are representative of two independent experiments (^*^ *p* < 0.05; ^**^ *p* < 0.01; ^***^ *p* < 0.001; ^****^ *p* < 0.0001).

We then asked whether IL-4Rα expression was influenced by *M. tuberculosis* infection in active TB and TB therapy cohorts. We extracted IL-4Rα and Foxp3 gene expression from the publicly available dataset (26) to understand the dynamics during the course of the disease in humans. The mRNA expression of the *Il4rα* gene significantly increased in the whole blood of active TB patients (aTB) compared to latently infected TB controls in the South African cohort (**Figure 7A**). In contrast, the increase in *foxp3* mRNA expression was significant yet moderate (**Figure 7B**). Interestingly, the increased expression of *Il4rα* in active TB patients reverted back to baseline levels upon the completion of anti-TB therapy (**Figure 7C**), while *foxp3* expression remained unaffected (**Supplementary Figure S5A**). This shows that IL-4Rα and Foxp3 T cells may play a role in the pathogenesis of human TB.

### Foxp3^cre^IL-4Rα^−/lox^ mice displayed similar lung bacterial loads, pathology, and survival during *M. tuberculosis* infection

To elucidate the role of IL-4Rα signalling on Foxp3 Treg cells, mice were infected with *M. tuberculosis* H37Rv at a low dose of 100 CFU/mouse by aerosol inhalation. Surprisingly, in contrast to *L. monocytogenes*, we found no survival benefit in these mice after 65 weeks of infection (**Figure 7D**). Consistently, Foxp3^cre^IL-4Rα^−/lox^ mice displayed similar mycobacterial burdens in the lungs and spleen (**Figure 7E, F**) and lung pathology and lesion size when compared to control animals at both 3 and 18 weeks postinfection (**Figures 7G, H**). This suggests that the deletion of IL-4Rα signalling on Foxp3 T regulatory cells has no effect on the outcome of *M. tuberculosis* infection.

### Foxp3^cre^IL-4Rα^−/lox^ mice influence lung immune responses during *M. tuberculosis* infection

We investigated whether the absence of IL-4Rα on Foxp3 Treg cells influenced the lung cytokine profile in tissue homogenates at 3 and 18 weeks post-infection (wpi). We found a significant decrease in nitric oxide (NO) (**Figure 7I**) and IL-10 production (**Figure 7J**) in Foxp3^cre^IL-4Rα^−/lox^ mice. IL-23 has been shown to contribute to the initiation of Th17 responses. While no differences in IL-17 production have been observed during BCG vaccination, IL-17 can contribute to pathological inflammation in the chronic stages of TB (45). We found a slight increase in IL-23 (stimulate IL-17 production) in the Foxp3^cre^IL-4Rα^−/lox^ mice at both time points (**Figure 7K**). GM-CSF production was significantly reduced in Foxp3^cre^IL-4Rα^−/lox^ mice (**Figure 7L**). While GM-CSF signalling is activated during *M. tuberculosis* infection, a study has shown that neutralising GM-CSF had no effect on the bacterial burden, however, a lack of GM-CSF led to increased granuloma formation in mice models (46). There was an increase in CXCL5, a neutrophil-activating chemokine, at 3 wpi in Foxp3^cre^IL-4Rα^−/lox^ mice (**Figure 7M**). Furthermore, no differences were observed in the levels of IL-1α, IL-4, IL-6, IL-12p40, IL-17, IFN-γ TNF, TGF-β, and GCSF (**Supplementary Figure S5B-J**), as well as the chemokines CXCL1, CXCL2, CCL3, and CXCL10 (**Supplementary Figure S5K-N**) at both time points. Overall, the absence of IL-4Rα signalling on Foxp3 Tregs has no major impact on lung cytokine and chemokine profiles, except for IL-23 and NO.

### IL-4Rα-mediated signalling on Foxp3 Tregs influences the immune cell populations and effector/memory T cells in lungs and lymph nodes in chronic *M. tuberculosis* infection

**Figure 8:**
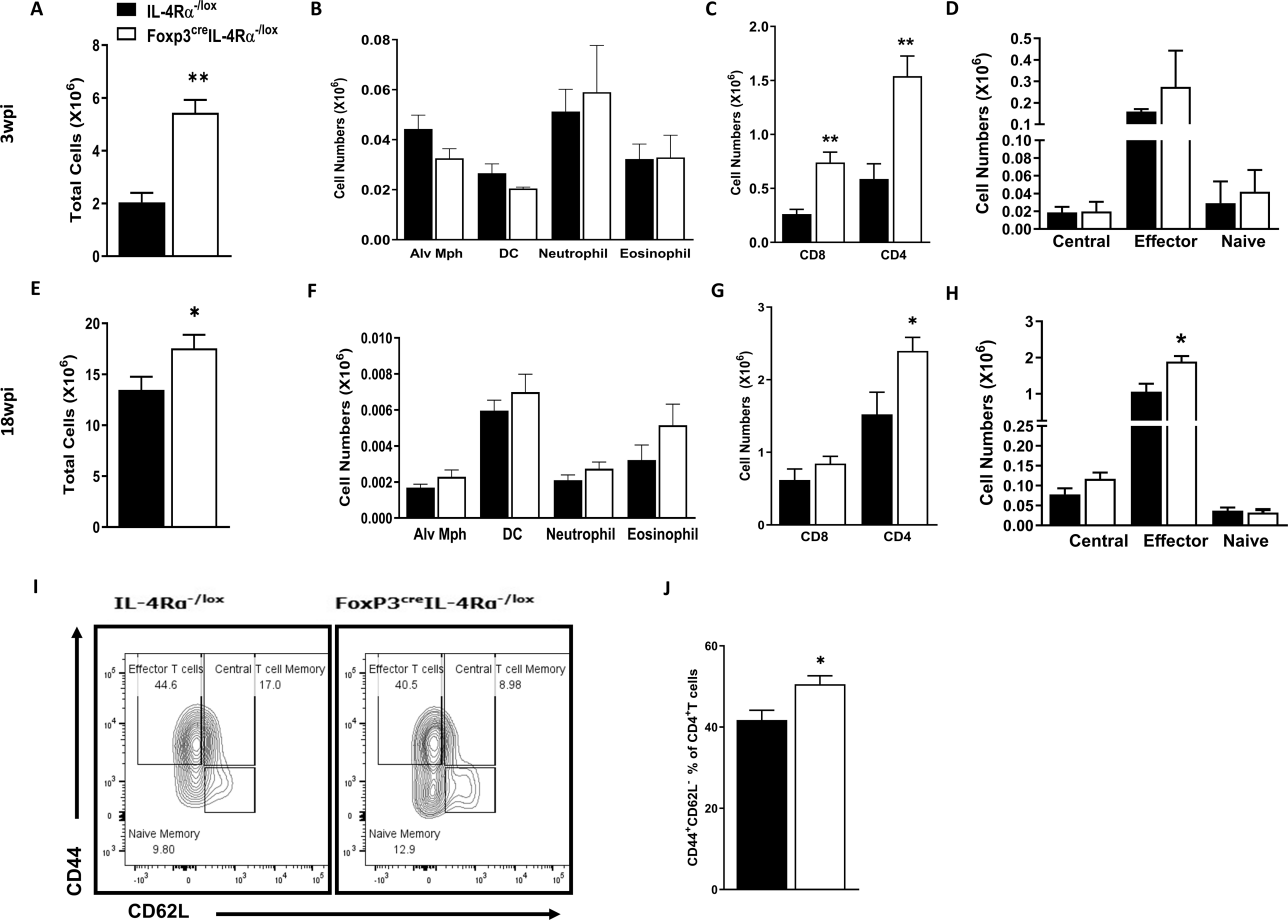
Increased T effector cells in the lungs during acute and chronic *M. tuberculosis* infection in Foxp3^cre^IL-4Rα^−/lox^ mice. Lung single-cell suspensions were prepared to determine myeloid and lymphoid populations at 3 and 18 weeks postinfection. **(A**, **E)** Total lung cell numbers; **(B**, **F)** Myeloid populations (alveolar macrophages, dendritic cells, neutrophils, and eosinophils); **(C**, **G)** CD4 and CD8 T cells; **(D**, **H)** CD4 T effector, naïve, and central cell numbers; **(I)** Representative flow plot of CD4 T effector, naïve, and central memory cells; and **(J)** Percentage of effector CD4 T cells. Data are represented as mean ± SEM of *n* = 5–6 mice/time, representative of two independent experiments, and analysed using the unpaired Student’s *t*-test (^*^ *p* < 0.05; ^**^ *p* < 0.01).

To further understand whether the abrogation of IL-4Rα signalling on Foxp3 Tregs alters the myeloid and lymphoid immune cell populations in the lungs at 3 and 18 wpi, we performed flow cytometry. There was a significant increase in total lung cell numbers in Foxp3^cre^IL-4Rα^−/lox^ mice, suggesting increased infiltration of immune cells at both 3 and 18 wpi (**Figures 8A, E**). Interestingly, this infiltration did not affect the myeloid (macrophages, dendritic cells, and neutrophils) populations (**Figures 8B, F**). However, both CD4 and CD8 T cells were significantly increased at 3 wpi (**Figure 8C**), while only CD4 T cells (**Figure 8G**) showed increased numbers at 18 wpi. We further investigated naïve, effector, and central memory CD4 T cells in the Foxp3^cre^IL-4Rα ^−/lox^ mice and found no difference in CD4 effector T cells at 3 wpi (**Figure 8D**). However, there was a significant increase in CD4 effector T cells (**Figure 8H**) at 18 wpi. Furthermore, we found a significant increase in the effector/effector memory phenotype (CD44^high^CD62L^low^) compared to controls (**Figures 8I, J**).

Given that lymphoid cells are enriched in the draining lymph nodes, we then investigated the impact of IL-4Rα signalling on Foxp3 Treg on the immune cell populations in the mediastinal lymph nodes at 3 and 18 wpi using flow cytometry. We observed a significant increase in total cell numbers in Foxp3^cre^IL-4Rα^−/lox^ mice, suggesting increased infiltration of immune cells into the lymph nodes at 3 wpi (**Supplementary Figure S6A**), but not at 18 wpi (**Supplementary Figure S6D**) during *M. tuberculosis* infection. Additionally, there was a significant increase in both CD4 and CD8 T-cell populations at both early and late infection (**Supplementary Figure S5B, E**). We also investigated whether the deletion of IL-4Rα on Treg affected naïve, effector, and central memory CD4 T cells in the Foxp3^cre^IL-4Rα^−/lox^ mice. Similar to the lungs, no significant differences were found in CD4 effector T cells in the Foxp3^cre^IL-4Rα^−/lox^ mice (**Figure 8C**) at 3 wpi. However, at 18 wpi, a significant increase in the central and effector CD4 T-cell memory in the Foxp3^cre^IL-4Rα^−/lox^ mice was observed (**Supplementary Figure S6F**). We also assessed whether these cells had augmented effector phenotype in the Foxp3^cre^IL-4Rα^−/lox^ mice at 18 wpi. Similar to the lungs, Foxp3^cre^IL-4Rα^−/lox^ mice had significantly higher effector/effector memory phenotype (CD44^high^CD62L^low^) compared to littermate controls in the lymph nodes (**Supplementary Figures S6G, H**). Altogether, these results suggest that the absence of IL-4Rα signalling on Tregs increases CD4 and CD8 T-cell recruitment, as well as effector T cells, in the lungs and lymph nodes during *M. tuberculosis* infection.

### IL-4Rα-mediated signalling on Foxp3 Tregs influences cytokine-producing CD4 T cells in lymph nodes during chronic *M. tuberculosis* infection

**Figure 9:**
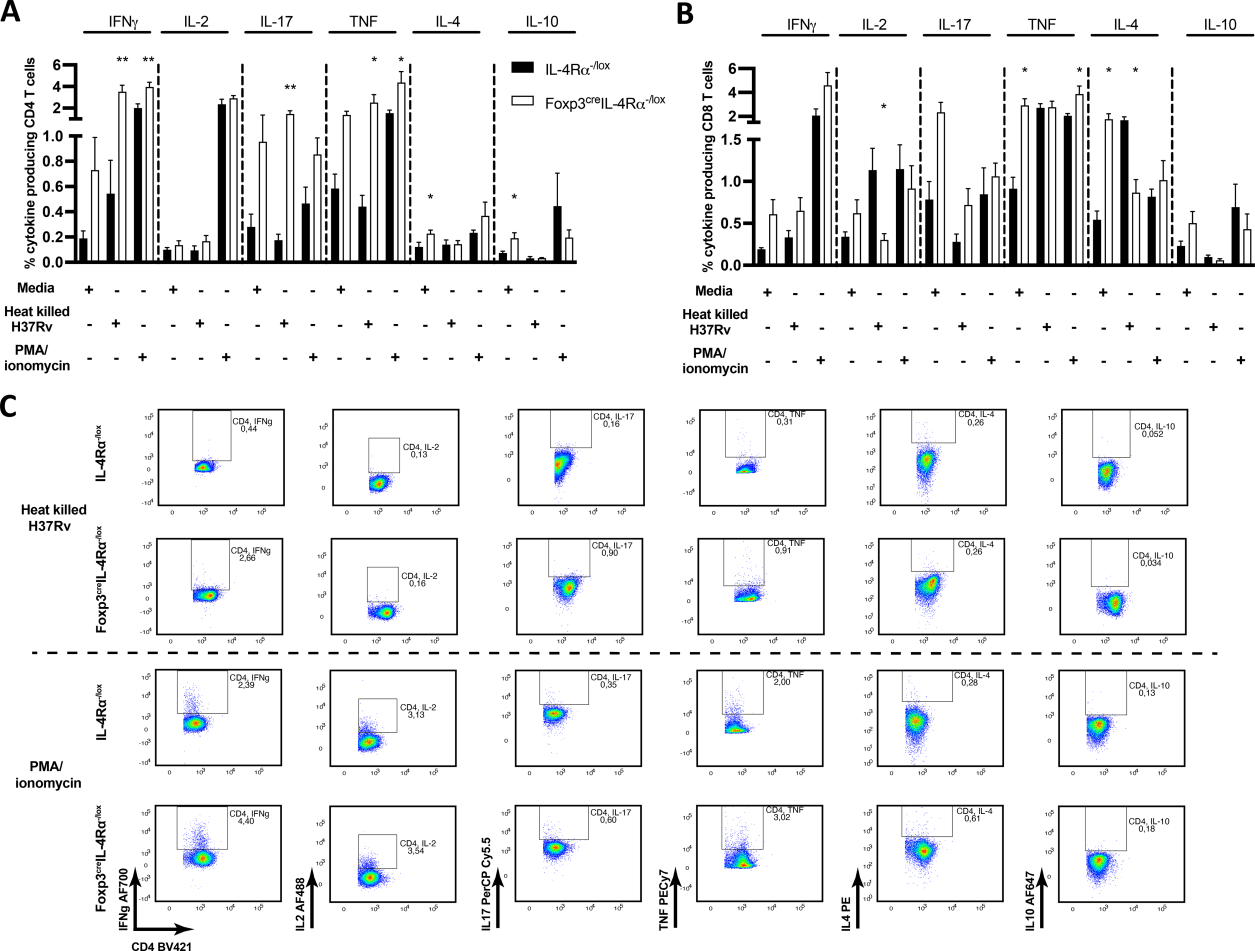
Increased CD4 T cells producing cytokines in the mediastinal lymph node of Foxp3^cre^IL-4Rα^−/lox^ mice during *M. tuberculosis* infection. A single-cell suspension of total mediastinal lymph nodes was prepared, and 2 million cells were then seeded to determine the frequency of the indicated cytokine production with heat-killed *M. tuberculosis* and PMA/ionomycin stimulation for 6 h, followed by monensin blockage to perform intracellular cytokine staining. **(A)** CD4 and **(B)** CD8 T-cell responses in unstimulated, heat-killed *M. tuberculosis* (H37Rv) and PMA/ionomycin stimulations. **(C)** Representative intracellular cytokine staining plots from H37Rv *M. tuberculosis* lysate and PMA/ionomycin-stimulated CD4 T cells. Data are represented as mean ± SEM of *n* = 5–6 mice/time, representative of two independent experiments, and analysed using the unpaired Student’s *t*-test (^*^ *p* < 0.05; ^**^ *p* < 0.01).

Given that we found no major differences in the lung cytokine and chemokine profiles (**Figure 7**; **Supplementary Figure S5**), we stimulated lymph node cells from 18-week *M. tuberculosis*-infected mice *ex vivo* to assess CD4 and CD8 T-cell-specific cytokines by intracellular cytokine staining. Cells were left unstimulated or stimulated with *M. tuberculosis* cell lysate (H37Rv) or PMA/ionomycin, followed by monensin secretion blockade. Foxp3^cre^IL-4Rα^−/lox^ mice showed no differences in IFN-γ, IL-2, IL-17, and TNF cytokine secretion in unstimulated CD4 (**Figure 9A**) and IFN-γ, IL-2, IL-17, and IL-10 in CD8 T cells (**Figure 9B**). However, IL-4 and IL-10 Th2 cytokine-secreting unstimulated CD4 T cells and TNF and IL-4 secreting CD8 T cells had higher percentages in the Foxp3^cre^IL-4Rα^−/lox^ group. As expected, with H37Rv lysate stimulation, CD4 T cells produced significantly higher levels of IFN-γ, IL-17, and TNF in Foxp3^cre^IL-4Rα^−/lox^ mice (**Figure 9A**), whereas IL-10-secreting CD4 and CD8 T cells decreased irrespective of groups (**Figures 9A, B**) and reduced IL-4-secreting CD8 T cells (**Figure 9B**). Importantly, CD4 T cells from the Foxp3^cre^IL-4Rα^−/lox^ group showed higher Th1/Th17 cytokine secretion upon H37Rv lysate or PMA/ionomycin stimulation (**Figures 9A–C**). Overall, these results suggest that the absence of IL-4Rα signalling on Tregs augments cytokine-producing CD4 T cells in the mediastinal lymph nodes during *M. tuberculosis* infection.

## Discussion

Foxp3 Tregs inhibit the activation and expansion of CD8 T cells, and their depletion leads to the expansion of CD8 T cells in *L. monocytogenes* infection (47). However, the role of IL-4Rα on Foxp3 Treg cells in *L. monocytogenes* infection has not been investigated. We found that IL-4Rα signalling is essential for maintaining Tregs and its loss leads to an increase in CD8 T cells in Foxp3^cre^IL-4Rα^−/lox^ mice. The quality of Foxp3 T cells is affected by signals from antigen and cytokine production (48). Remarkably, the depletion of Foxp3 Tregs resulted in host protection in *L. monocytogenes* and *Salmonella enterica* infections (20, 49, 50). Indeed, the quality of Foxp3 Tregs was decreased in Foxp3^cre^IL-4Rα^−/lox^ mice, signifying that IL-4Rα is required to maintain the stability, and probably the function, of Tregs in *L. monocytogenes* infection. The decrease of Foxp3 Tregs tilts the immune balance toward an effector Th1 phenotype, which is fundamental for *L. monocytogenes* clearance (51). Remarkably, a decrease in the expression of Foxp3 in the spleen was observed, which was not affected by the loss of IL-4Rα at a steady state. A multigene and fate-reporter system demonstrated that the shift from Foxp3 Tregs to exFoxp3 (Th2) is IL-4-dependent during *Heligmosomoides polygyrus* infection and allergy (52). Foxp3 instability has been shown to transiently polarize Foxp3 T cells into Th2 (exFoxp3 T cells), which then proliferate and produce inflammatory cytokines in tissues (53). Consistently, the loss of IL-4Rα on Foxp3, which led to increased T-bet expression, suggests an enhanced Th1 response in Foxp3^cre^Il-4Rα^−/lox^ mice. In addition, we demonstrated that the lack of Treg stability was due to the loss of IL-4Rα, which resulted in a significant increase in effector T cells increased during *L. monocytogenes* infection, thereby asserting the imbalance observed in earlier studies. The Foxp3 regulatory T-cell population is also known to impede the priming of effector T cells (47, 54–56). This interplay is fundamental, as the ratio of Tregs to effector immune cells is critical to shaping the host response (57).

Tregs reduce the CD4 T-cell-dependent cytotoxic CD8 T-cell response to *L. monocytogenes*, impeding the body’s ability to control infection (58). In mice, transient ablation of Foxp3 Treg causes increased CD8 T cells, enhancing protection against *L. monocytogenes* (20). CD8 T cells play a very crucial role in *L. monocytogenes* infection (19, 31, 34, 35, 59), exerting effector fuctions such as secreting granzyme B and perforin (51). IFN-γ, as an effector cytokine, provides protective effects that extend beyond the direct action of granzyme B (60). We found that targeting IL-4Rα led to higher secretion of granzyme B and increased IFN-γ levels in the serum. The increase in IFN-γ further correlated with T-bet expression (with no change in GATA3) in CD8 T cells of Foxp3^cre^IL-4Rα^−/lox^ mice. Moreover, these cells showed increased proliferation (Ki-67), enhanced survival (Bcl2), and a concomitant decrease in apoptosis (caspase 3) in Foxp3^cre^IL-4Rα^−/lox^ mice. However, the latter is in contrast with a study indicating that exogenous treatment of Tregs with IL-4 has an anti-apoptotic effect (61). Increased IFN-γ levels have been shown to correlate with the clearance of *L. monocytogenes* in the spleen and peritoneal cavity, with early release from NK cells followed by T cells (29, 62, 63). Foxp3^cre^IL-4Rα^−/lox^ mice showed no differences at earlier stages, but showed a significant increase in IFN-γ production by T cells at later time points when stimulated *ex vivo*. IL-2 controls the expression of transcription factors, thereby contributing to Th1 cell development (64), while also playing a critical role in maintaining Tregs, facilitating T-cell differentiation, and controlling cytokine secretion (65–67). Consistent with this, IL-2 levels were increased concomitantly with IFN-γ upon stimulations in the Foxp3^cre^IL-4Rα^−/lox^ mice during *L. monocytogenes* infection, correlating with effector and Th1 cytokines with conspicuous T-bet expression.

The splenic myeloid cells in the marginal zone of the red pulp increase protection (59, 68), but the T-zone of the white pulp is where effector T cells cross-talk with dendritic cells (69, 70). The depletion of white pulp indicated a critical role of myeloid cell interactions with effector CD8 T cells during *L. monocytogenes* infection (71). In Foxp3^cre^IL-4Rα^−/lox^ mice, reduced destruction of white pulp suggests improved control during *L. monocytogenes* infection. This outcome may be partially a result of the increase in neutrophils, dendritic cells, and inflammatory macrophages early after infection in the spleen. This finding is consistent with reports showing that early control of *L. monocytogenes* is mediated by the neutrophils and macrophages (30, 72, 73). Our findings revealed that IL-4Rα signalling on Tregs plays a tissue-destructive role during *L. monocytogenes* infection.

On the other hand, Foxp3^cre^IL-4Rα^−/lox^ mice displayed similar bacterial loads and lung pathology during both acute and chronic *M. tuberculosis* infection, suggesting a varying role of IL-4Rα signalling. However, Foxp3^cre^IL-4Rα^−/lox^ mice showed increased numbers of both CD4 and CD8 T-cells, suggesting a shift the T-cell balance. Moreover, CD4 but not CD8 T cells from Foxp3^cre^IL-4Rα^−/lox^ mice produced increased levels of IFN-γ, TNF, and IL-17, indicating the ability of IL-4Rα signalling on Tregs to modulate CD4 T-cell immune responses during *M. tuberculosis* infection. This finding is consistent with previous observations that while CD8 T cells are required, they are not as indispensable as CD4 T cells in *M. tuberculosis* infection (74). Given that the Foxp3^cre^IL-4Rα^−/lox^ mice showed a larger proportion of functional *M. tuberculosis*-specific CD4 T cells with no changes in bacterial burden and lung pathology, it is plausible that there may be a potential compensation of IL-4Rα signalling by other immune cells. Our research revealed no discernible variations in T-cell-specific cytokines across the groups in the CD4 and CD8 T cells of the draining lymph nodes. The fact that T-cell-specific cytokines were not measured in the lungs limits the study. However, our laboratory’s work using the same Foxp3^cre^IL-4Rα^−/lox^ mice in an allergic asthma model revealed that the profiles of CD4 T cells producing IL-17 and IL-10 in both the lymph nodes and lungs were similar (75). This work also showed that Tregs capacity to reduce type 2 cytokine production in IL-C2a- and IL-33-mediated inflammation was inhibited by IL-4Rα signalling in an IL-10-dependent way. Previously, we showed the importance of IL-4Rα signalling on other immune cells, such as B cells, which increased host protection in chronic TB (25). In contrast, IL-4Rα signalling on macrophages/neutrophils resulted in marginal susceptibility to TB (76).

Overall, we provide a new perspective on the Treg-specific function of IL-4Rα signalling in listeriosis, highligting the importance of the stability and quality of Foxp3 Tregs. The absence of IL-4 signalling in the Treg population led to the production of increased proinflammatory cytokines, including IFN-γ, which are essential for *L. monocytogenes* clearance. In *M. tuberculosis* infection, while the CD4 T effector cytokines were altered in the absence of IL-4Rα on Tregs in the lymph nodes, this did not affect the outcome of TB disease in mice. Together, we reveal an unappreciated biological function of IL-4Rα signalling in regulating the differential roles of Tregs in listeriosis and tuberculosis.

## Data Availability

The raw data supporting the conclusions of this article will be made available by the authors, without undue reservation.

## Supplementary material

Supplementary Figure 1 Gating strategy for the identification of IL-4Rα expression and Foxp3 cells in the liver and spleen of the mice.

Supplementary Figure 2 Gating strategy activation and memory cell populations and intracellular cytokine staining. **(A)** Gating strategy representing the identification of memory and activation status of lymphoid cell population in the liver and spleen of the mice. **(B)** Gating strategy shows intracellular cytokine assay staining.

Supplementary Figure 3 Gating strategy Lymphoid and Myeloid cell populations in the indicated tissues. **(A)** Gating strategy representing the identification of lymphoid cell population in the liver, spleen and lung of the mice. **(B)** Gating strategy shows myeloid cell populations in the liver.

Supplementary Figure 4 Gating strategy Myeloid cell populations. **(A)** Gating strategy representing the identification of myeloid cell populations in the spleen. **(B)** Gating strategy shows myeloid cell populations in the lungs.

Supplementary Figure 5 Cytokine/chemokine profile in the lungs of *Mtb*-infected Foxp3^cre^IL-4Rα^-/lox^ mice. **(A)** Foxp3 expression profile during treatment in the South African cohort. **(B)** IL-1α, **(C)** IL-4, **(D)** IL-6, **(E)** lL-12p40, **(F)** IL-17, **(G)** TNF, **(H)** TGF-β, **(I)** GM-CSF, **(J)** G-CSF, **(K)** CXCL1, **(L)** CXCL2, **(M)** CXCL10 and **(N)** CCL3 measured by ELISA. Data represented as mean± SEM of n= 5-6 mice/group from two independent experiments and analysed using unpaired, student t-test.

Supplementary Figure 6 Foxp3^cre^IL-4Rα^-/lox^ mice increased T effector cells in the lymph nodes during acute and chronic *Mtb* infection. Single cell suspensions from the mediastinal lymph nodes were prepared to determine lymphoid populations at 3 and 18 weeks post infection. **(A, D)** Total lymph node cell numbers, **(B, E)** CD4 and CD8 T cells **(C, F)** CD4 T effector, naïve and central cell numbers. **(G)** Represented flow plot of CD4 T effector, naïve and central memory cells and **(H)** Percentage of effector CD4 T cells. Data represented as mean± SEM of n=5-6 mice/time representative of two independent experiments and analysed using unpaired, student t-test. (**p <*0.05, ***p <*0.01.
