# Supplementary figures and images for: The divergent outcome of IL-4Rα signalling on Foxp3 T regulatory cells in listeriosis and tuberculosis
Source: Front Immunol. 2024 Oct 17;15:1427055. doi: 10.3389/fimmu.2024.1427055 (PMC11524857; doi:10.3389/fimmu.2024.1427055)

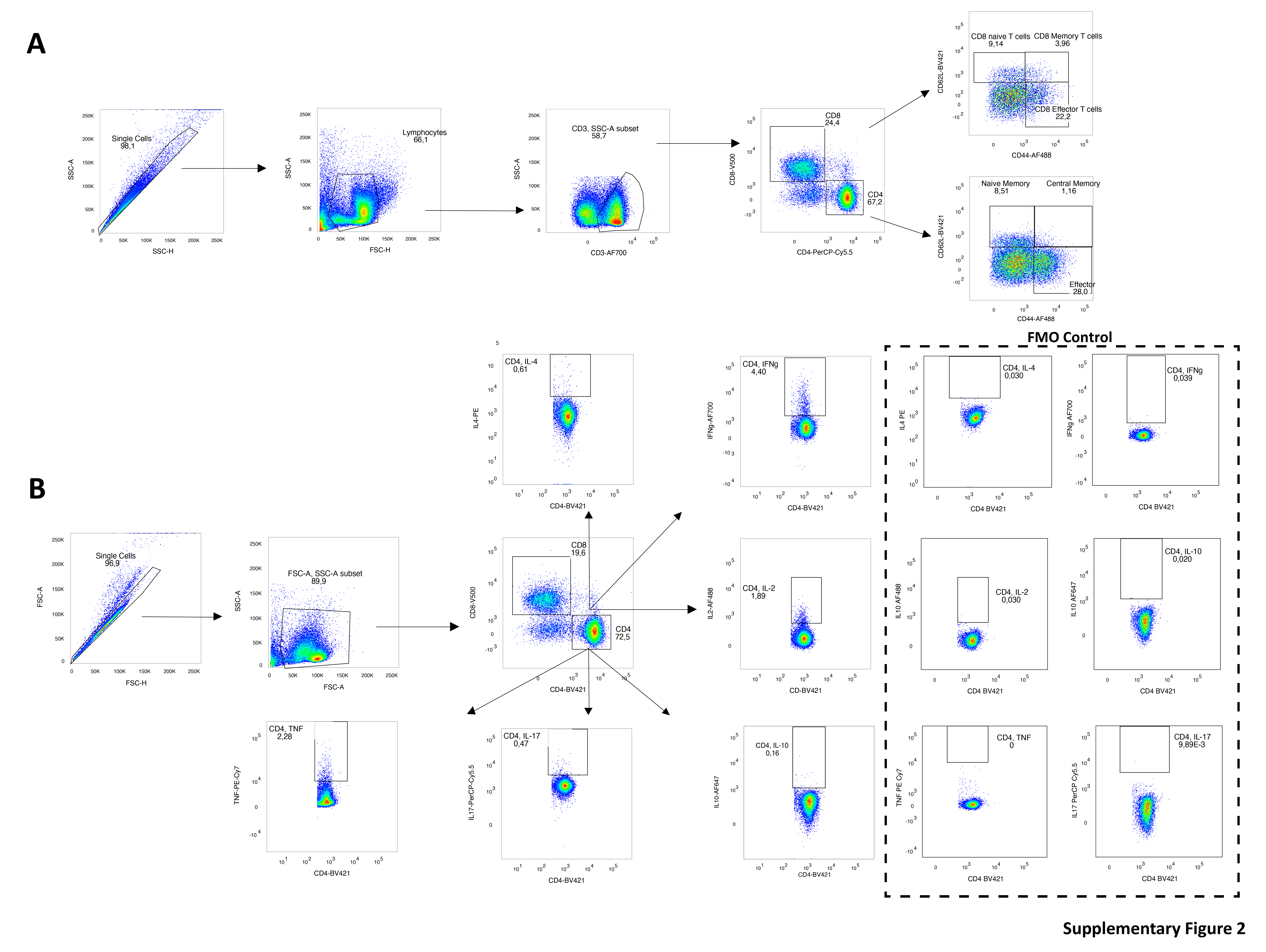

Supplement: Supplementary Figure 5 — Cytokine/chemokine profile in the lungs of Mtb-infected Foxp3creIL-4Rα-/lox mice. (A) Foxp3 expression profile during treatment in the South African cohort. (B) IL-1α, (C) IL-4, (D) IL-6, (E) lL-12p40, (F) IL-17, (G) TNF, (H) TGF-β, (I) GM-CSF, (J) G-CSF, (K) CXCL1, (L) CXCL2, (M) CXCL10 and (N) CCL3 measured by ELISA. Data represented as mean± SEM of n= 5-6 mice/group from two independent experiments and analysed using unpaired, student t-test. [file Image5.tif]

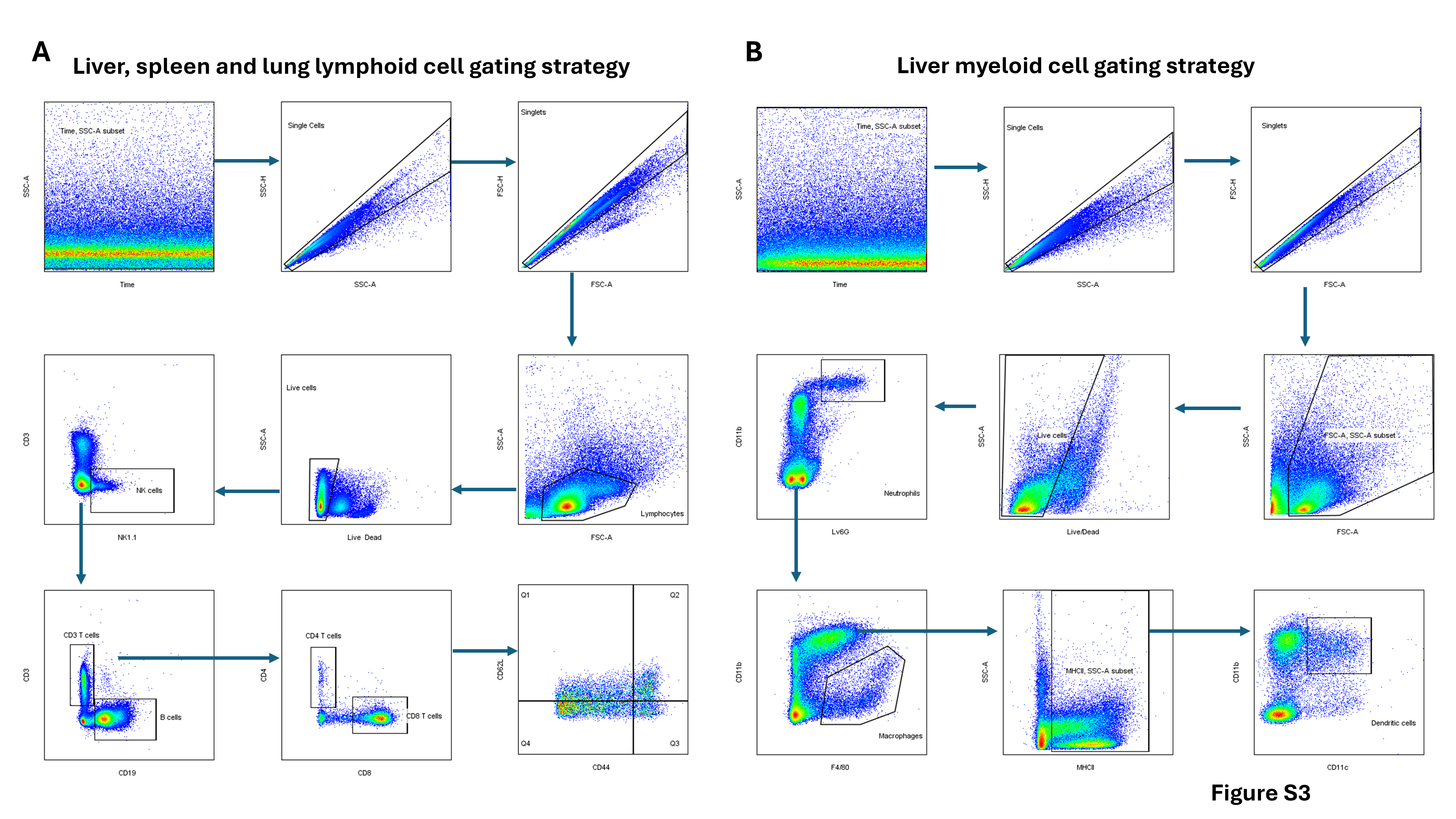

Supplement: Supplementary Figure 6 — Foxp3creIL-4Rα-/lox mice increased T effector cells in the lymph nodes during acute and chronic Mtb infection. Single cell suspensions from the mediastinal lymph nodes were prepared to determine lymphoid populations at 3 and 18 weeks post infection. (A, D) Total lymph node cell numbers, (B, E) CD4 and CD8 T cells (C, F) CD4 T effector, naïve and central cell numbers. (G) Represented flow plot of CD4 T effector, naïve and central memory cells and (H) Percentage of effector CD4 T cells. Data represented as mean± SEM of n=5-6 mice/time representative of two independent experiments and analysed using unpaired, student t-test. (*p <0.05, **p <0.01. [file Image6.tif]
